# An equation to estimate 24-hour total urine protein excretion rate in patients who underwent urine protein testing

**DOI:** 10.1186/s12882-022-02673-2

**Published:** 2022-01-29

**Authors:** Fan Yang, Jing-Song Shi, Si-Wen Gong, Xiao-Dong Xu, Wei-Bo Le

**Affiliations:** 1 National Clinical Research Center of Kidney Diseases, Jinling Hospital, Nanjing University School of Medicine, Zhongshan East Street 305, Nanjing, China; 2 National Clinical Research Center of Kidney Diseases, Jinling Hospital, the First School of Clinical Medicine, Southern Medical University, Nanjing, China

**Keywords:** Urine protein-creatinine ratio, Proteinuria, Prediction model

## Abstract

**Background:**

The urine protein-creatinine ratio (UPCR) in a spot first-morning urine sample is used to estimate 24-h urine proteinuria (24hUP) in patients who underwent urine protein testing. UPCR cannot be directly compared with 24-h proteinuria. Thus, an equation to estimate 24-h total protein excretion rate, using age, gender, and the UPCR may improve its bias and accuracy in patients who underwent urine protein testing.

**Methods:**

We simultaneously measured 24-h urine protein and the same day’s first-morning spot urine from patients with kidney disease. Generalized linear and no-linear models, using age, gender, and UPCR, were constructed to estimate for 24-h urine protein and the best model (NJ equation) was selected to estimated 24 hUP (e24hUP).

**Results:**

A total of 5435 paired samples (including a training cohort of 3803 patients and a validation cohort of 1632 patients) were simultaneously measured for UPCR and 24-h urine protein. In the training cohort, the unadjusted UPCR obviously underestimated 24-h urine protein when UPCR ≤1.2 g/g (median bias − 0.17 g/24 h) and overestimated 24-h urine protein when UPCR > 1.2 g/g (median bias 0.53 g/24 h). In the validation cohort, the NJ equation performed better than the unadjusted UPCR, with lower root mean square error (0.81 vs. 1.02, *P* < 0.001), less bias (median difference between measured and estimated urine protein, − 0.008 vs. 0.12), improved precision (interquartile range of the differences, 0.34 vs. 0.50), and greater accuracy (percentage of estimated urine protein within 30% of measured urine protein, 53.4% vs. 32.2%). Bland-Altman plot indicated that the agreement of spot and daily estimates was less pronounced with 24 hUP > 2 g than lower values.

**Conclusions:**

The NJ e24hUP equation is more accurate than unadjusted UPCR to estimate 24 hUP in patients with kidney disease and could be used for laboratory application.

**Supplementary Information:**

The online version contains supplementary material available at 10.1186/s12882-022-02673-2.

## Background

Proteinuria is a common feature of chronic nephropathies, and those with greater levels of proteinuria are at greater risk of declining in glomerular filtration rate, as well as progression to end-stage renal disease (ESRD) [1]. Quantitative assessment of urinary protein excretion is critical in patients with proteinuric kidney disease. Urinary protein excretion rate has been traditionally measured using 24-h urine collections. The 24-h urine protein (24hUP) is considered as the gold standard for measurement of protein excretion in patients with proteinuric kidney disease. However, a 24-h urine collection is cumbersome for patients and frequently collected incorrectly. Such collection errors are problematic because the error in estimating 24-h proteinuria from the protein content of intended 24-h collections is directly proportional to the extent of over collection or under collection [2, 3]. Moreover, collection of 24-h urine in the hospital can cause spread of some species of bacteria, such as multidrug-resistant *Pseudomonas aeruginosa*, which causes urinary tract infection [4, 5]. As previous studies indicated, the urine protein-creatinine ratio (UPCR) in a single spot first-morning urine sample, could serve as a convenient and satisfactory substitute for the determination of protein excretion in 24-h urine collection [6, 7].

Although UPCR is recognized as a screening tool for patients with proteinuric kidney diseases, the raw unadjusted UPCR cannot be directly compared with 24-h proteinuria in patients with proteinuric kidney diseases. The urine protein and creatinine excretion can vary throughout the day and UPCR is remarkably influenced by the urine creatinine excretion [8–11]. Several equations which adjust gender, age and/or other variables to estimate 24 hUP may improve its bias and accuracy in patients with proteinuric kidney disease [12–14]. However, the relationship between UPCR and 24 hUP may differ across races and clinical centers. In this study, we assessed the correlation between first-morning spot UPCR and 24 hUP from 5435 paired urine samples with diverse degrees of proteinuria. We developed an equation (Named NJ equation) to estimate 24 hUP based on UPCR, age, and gender in our renal center.

## Methods

### Study subjects and laboratory measurements

Patients who had visited National Clinical Research Center of Kidney Diseases, Jinling Hospital between May of 2019 and December of 2020, were screened in this study. A total of 5526 patients with both intact UPCR and 24 hUP test results were referred. Exclusion criteria included the following: (1) being aged < 18 years; (2) 24 h urine amount < 400 mL; (3) having UPCR > 15 g/g or 24 hUP > 15 g. Finally, 5435 patients were enrolled for data analysis. The whole cohort was randomly divided into two cohorts with a ratio of 7:3, namely the training cohort (3803 patients) and the validation cohort (1632 patients).

All patients were instructed to begin collecting urine in the container and to collect all urine continuously for 24 h. All examination experiments were conducted in the central laboratory at our hospital. The first-morning midstream urine sample was collected to test UPCR. The volume of collected 24-h urine was measured by the laboratory staff, and protein level in samples taken from 24-h urine collections was determined using standard laboratory methods in the central laboratory at our hospital (Cobas Integra analyzers, Roche). The spot urine specimen was assayed for urine protein concentration and urine creatinine concentration. The UPCR was calculated by dividing the urine protein concentration by the creatinine concentration.

### Equation derivation and equation performance

UPCR was log transformed or square-root transformed. The form with better correlation coefficient with 24 hUP was chosen in the candidate 24 hUP estimation equations. Several candidate equations were derived using least squares regression. Age and gender could be included in the candidate equations, while body surface area indexing (BSA) was not included in the equations for BSA is often unavailable in laboratory application. Generalized linear smooths (glm) or local smooths (loess) were used to observe the relationship between 24 hUP and predictive variables. In general, the model with the smallest RMSE (root mean square error) was preferred and was carried forward in the validation cohort. Interactions for square-root transforming UPCR and gender, as well as for square-root transforming UPCR and age, were also included in the regression model if they were significant (*P* < 0.001) and improved model performance (with lower RMSE).

Bias (Median difference), precision, and accuracy were calculated to determine equation performance, as proposed in other similar studies [15, 16]. Bias was defined as the median difference between the measured 24 hUP (m24hUP) and the e24hUP, while precision, reflected by an IQR (interquartile range) of this difference. A positive value of bias indicates that the equation overestimates 24 hUP, and a negative value indicates underestimation. Accuracy was assessed as the RMSE relative to m24hUP. P30 is defined as the percentages of individuals that are within 30% difference from the m24hUP. Bootstrap methods (2000 bootstraps) were used to compute 95% CIs for these four equation performances. The degree of agreement was validated by Bland-Altman analysis. We used the area under receiver-operating characteristic curve (AUROC) at different specified thresholds (> 0.15, > 0.5, > 3.5 g/24 h) according to the thresholds recommended by KDIGO to measure the diagnostic value of NJ equation [1]. After the best Youden’s index was calculated, the according sensitivity, specificity, positive likelihood ratio, and negative likelihood ratio were identified. Accuracy was defined as the proportion of patients correctly classified according to unadjusted UPCR and e24hUP calculated by NJ equation above specific m24hUP thresholds in the entire ROC analysis.

Kolmogorov-Smirnov test was conducted to determine whether variables involved in our study were normally distributed. Normally distributed variables are expressed as mean ± sd. and were compared using Student’s t-test or one-way analysis of variance. Skewedly distributed variables are expressed as median and range and compared using either Mann–Whitney U test or Kruskal–Wallis H test. Categorical variables are expressed in percentages and compared using the Pearson χ2 test or Fisher’s exact test. Correlation between two variables were determined by Pearson correlation test. Two-tailed *P* < 0.01 findings were considered statistically significant. There were no missing data in this study. All analyses were carried out using R version 4.0.2 (Free Software Foundation, Boston, Massachusetts).

## Results

### Clinical and demographic characteristics of the study population

From May 2019 to December 2020, 5435 patients who visited National Clinical Research Center of Kidney Diseases, Jinling Hospital between were enrolled in this study. The whole cohort was randomly divided into the training cohort and the validation cohort with a ratio of 7:3. Table 1 shows the demographic and clinical characteristics of these two cohorts. The median age of patients in the training and validation cohort were 48 (lower quartile - upper quartile: 36–56) vs 48 years (lower quartile - upper quartile: 36–56) (*P* = 0.676). In the training cohort, median 24 hUP was 0.63 g/24 h (lower quartile - upper quartile: 0.34–1.32), with no significant difference from the validation cohort (0.62 g/24 h, lower quartile - upper quartile:0.34–1.37, *P* = 0.701). Similarly, there is no significant difference between these two cohorts in gender ratio, urine protein, urine creatinine, UPCR, and 24 h urine amount (*P* = 0.947, 0.856, 0.707, 0.854, and 0.926, respectively).

**Table 1:** Demographic and clinical characteristics of patients in the training and validation cohort

Variables	Total (*n* = 5435)	Training cohort (*n* = 3803)	Validation cohort (*n* = 1632)	*P*
Age, Median (IQR), y	48 (36, 56)	48 (36, 56)	48 (36, 56)	0.676
Gender, n(%)				0.947
Female	2593 (47.7)	1816 (47.8)	777 (47.6)	
Male	2842 (52.3)	1987 (52.2)	855 (52.4)	
UP, Median (IQR), g/L	0.57 (0.16, 1.66)	0.56 (0.16, 1.67)	0.58 (0.15, 1.65)	0.856
UCRE, Median (IQR), g/L	1.18 (0.77, 1.77)	1.17 (0.77, 1.79)	1.19 (0.76, 1.74)	0.707
UPCR, Median (IQR), g/g	0.49 (0.14, 1.49)	0.49 (0.14, 1.49)	0.49 (0.14, 1.49)	0.854
24hUA, Median (IQR), mL	1800 (1400, 2300)	1800 (1400, 2300)	1800 (1400, 2300)	0.926
24hUP, Median (IQR), g/24 h	0.62 (0.34, 1.33)	0.63 (0.34, 1.32)	0.62 (0.34, 1.37)	0.701

*IQR* interquartile range, *UP* urine protein, *UCRE* urine creatinine, *UPCR* urine protein-creatinine ratio, *24 hUA* 24-h urine amount, *24 hUP* 24-h urine protein

### Correlations between UPCR and 24-h urine protein (24hUP) in the training cohort

**Fig. 1:**
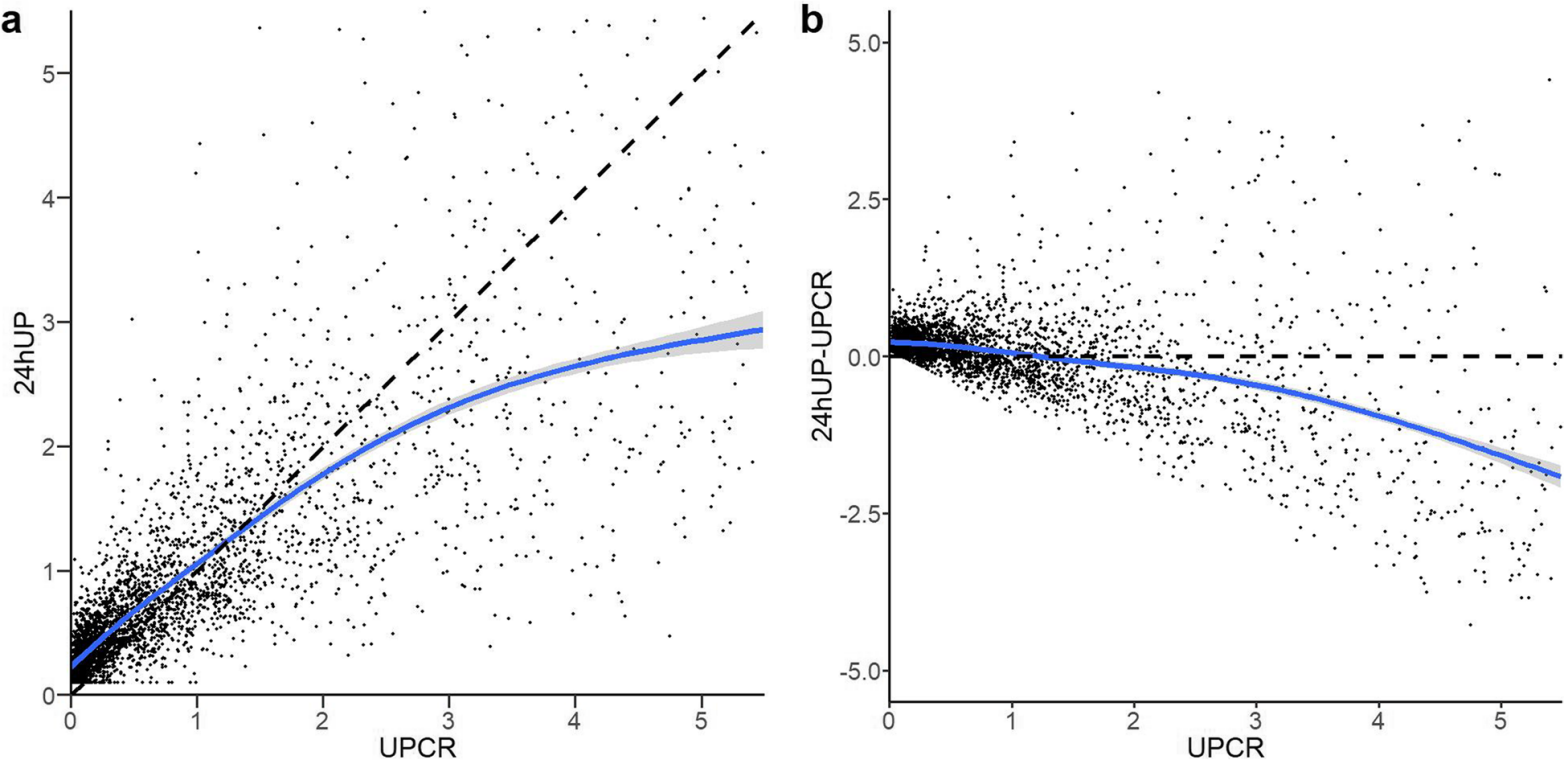
Scatter plot of 24 hUP and UPCR (**a)** /24 hUP-UPCR (**b**). 24 hUP, 24-h urine protein; UPCR, urine protein-creatinine ratio

There was a strong correlation between UPCR and 24 hUP in the training cohort (Pearson correlation coefficient = 0.85) (Fig. 1a). Overall, the UPCR underestimated the 24 hUP (bias = − 0.12 g/24 h). More precisely, the UPCR underestimated 24 hUP when UPCR ≤1.2 g/g (bias = − 0.17 g/24 h), but obviously overestimated 24 hUP when UPCR > 1.2 g/g (bias = 0.53 g/24 h) (Fig. 1b). In the training cohort, UPCR and 24 hUP were strongly correlated when patients were stratified by age (Pearson correlation coefficient = 0.85 for patients < 48 years old and 0.86 for patients ≥48 years old, respectively)and gender (Pearson correlation coefficient = 0.87 for male and 0.84 for female, respectively) (Supplementary Table 1).

### Construction of the equations to estimate 24 hUP

Square-root transformed UPCR and 24 hUP strengthened the Pearson correlation coefficient to 0.88, better than the log-transformed ones (Pearson correlation coefficient = 0.86). Thus, UPCR and 24 hUP were both transformed into square-root scale in the following analysis. Several candidate equations were developed to estimate 24 hUP (Table 2). We focused on RMSE to select the best model. Among the models, the model 4, named as NJ equation, showed the best performance, with the lowest RMSE in the training cohort. Although both the interaction between square-root transformed UPCR and age (*P* < 0.001) and interaction between square-root transformed UPCR and gender (*P* < 0.001) were significant in the model 4, involvement of either or both of them in the model 4 failed to decrease RMSE. Thus, the model without interactions was carried forward for further analysis. The exact model 4 (the NJ equation) for estimating 24 hUP in a Chinese population as follows:

**Table 2:** Candidate Regression Models for 24 hUP and RMSE value in the training cohort

ID	Models	RMSE
0	UPCR	1.10
1	β_0_ + β_1_ × UPCR	0.91
2	(β_0_ + β_1_× $\sqrt{\mathrm{UPCR}}$square root of UPCR)^2^	0.93
3	(β_0_ + β_1_× $\sqrt{\mathrm{UPCR}}$square root of UPCR + β_2_× gender + β_3_ × age)^2^	0.89
4	(β_0_ + β_1_× $\sqrt{\mathrm{UPCR}}$square root of UPCR + β_2_× gender + β_3_ × age + β_4_ × UPCR) ^2^	0.88
5	(β_0_ + (β_1_ + β_2_ × gender + β_3_ × age)× $\sqrt{\mathrm{UPCR}}$square root of UPCR)^2^	0.88

*UPCR* urine protein-creatinine ratio

For Female:

$${\left(0.623\times \sqrt{UPCR}+0.0323\times UPCR-0.0024\times age+0.381\right)}^2$$

For Male:

$${\left(0.623\times \sqrt{UPCR}+0.0323\times UPCR-0.0024\times age+0.522\right)}^2$$

### Performance of the NJ equation

As shown in Table 3, the NJ equation in the training cohort improved median bias compared with unadjusted UPCR (− 0.010 g/24 h vs. 0.12 g/24 h, *P* < 0.001), as well as IQR (0.36 g/24 h vs. 0.35 g/24 h, *P* < 0.001), P30 (52.5% vs. 31.0%, *P* < 0.001), and RMSE (0.88 vs. 1.10, *P* < 0.001).

**Table 3:** Comparison of the UPCR and NJ Equations in estimating measured 24-h urine protein

Variable and Equation	Training cohort	Validation cohort
RMSE (95% CI)		
UPCR	1.10 (1.03, 1.17)	1.02 (0.91, 1.12)
NJ Equation	0.88 (0.82, 0.94)	0.81 (0.73, 0.89)
Bias (95% CI)		
UPCR	0.12 (0.11, 0.13)	0.12 (0.10, 0.13)
NJ Equation	−0.010 (−0.020, −0.001)	−0.008 (−0.022, 0.004)
IQR (95% CI)		
UPCR	0.46 (0.43, 0.49)	0.47 (0.41, 0.53)
NJ Equation	0.35 (0.33, 0.36)	0.34 (0.30, 0.36)
P30(%) (95% CI)		
UPCR	31.0 (29.4, 32.6)	32.2 (29.9, 34.5)
NJ Equation	52.5 (50.8, 54.0)	53.4 (50.7, 56.1)

*RMSE* root mean square error, *CI* confidence interval, *UPCR* urine protein-creatinine ratio, *IQR* interquartile range, *P30* percentages of individuals that are within 30% difference

RMSE, Bias, IQR and P30 were all presented as values (95% confidence intervals).

**Fig. 2:**
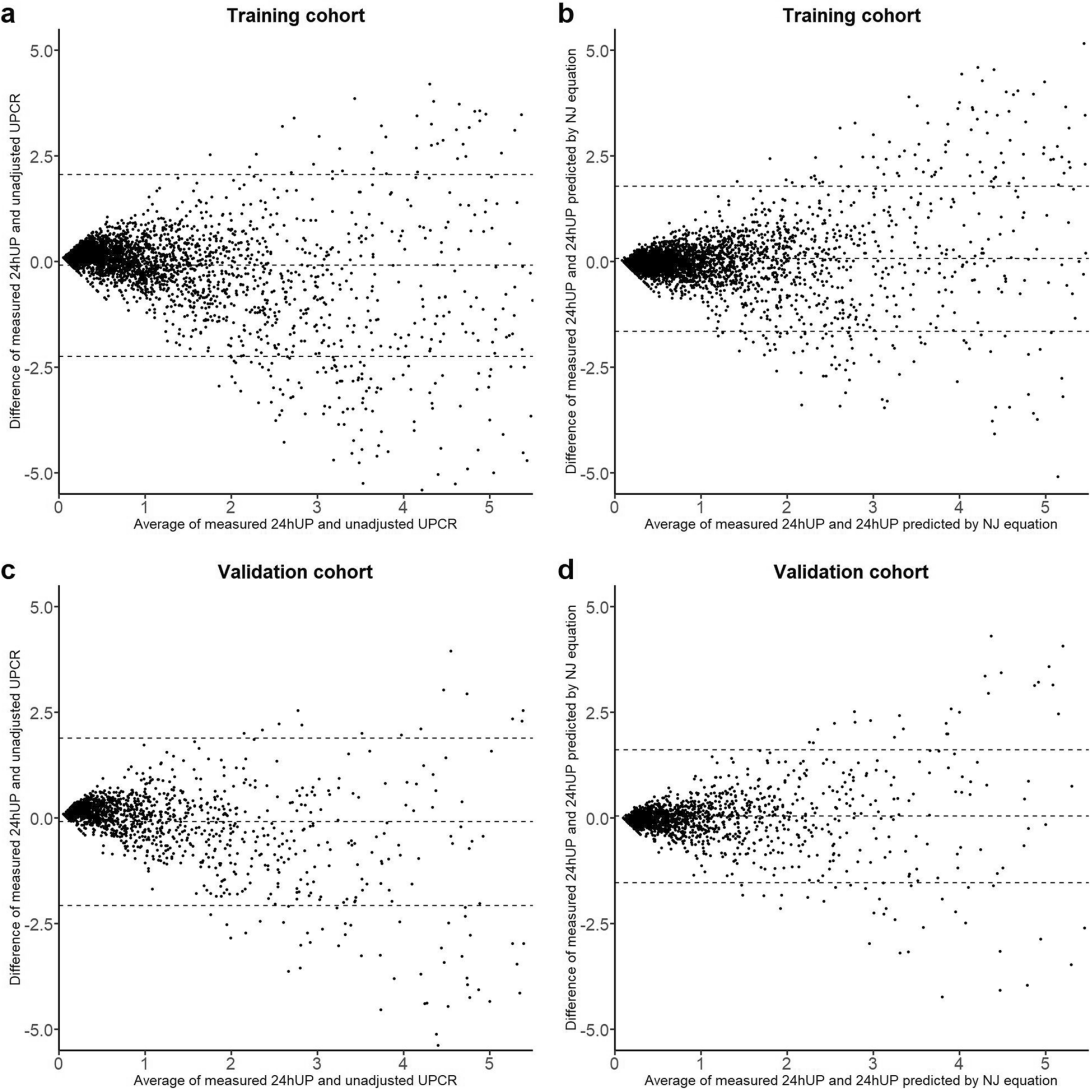
**a** Bland-Altman plot of measured 24 hUP and UPCR in the training cohort. **b** Bland-Altman plot of measured 24 hUP and predicted 24 hUP by NA equation in the training cohort. **c** Bland-Altman plot of measured 24 hUP and UPCR in the validation cohort. **d** Bland-Altman plot of measured 24 hUP and predicted 24 hUP by NA equation in the validation cohort. UPCR, urine protein-creatinine ratio. The solid lines represent the mean difference, and the dotted lines represent the 95% CI limits of agreement

In the validation cohort, the NJ equation also had significantly lower RMSE (0.81 vs. 1.02, *P* < 0.001), lower median bias (− 0.008 g/24 h vs. 0.12 g/24 h, *P* < 0.001), lower IQR (0.34 g/24 h vs. 0.47 g/24 h, *P* < 0.001), and higher P30 (53.4% vs. 32.2%, *P* < 0.001) than unadjusted UPCR. As shown by the Bland–Altman plot, the 95% CI limit of agreement between spot estimated 24 hUP (by NJ equation) and measured 24 hUP were narrower than that between unadjusted UPCR and measured 24 hUP, both in the training and validation cohort (Fig. 2). Bland-Altman plot also indicated that the agreement of spot and daily estimates was less pronounced with 24 hUP > 2 g than lower values, both in the training and validation cohort (Fig. 2). Stratified analysis of the validation cohort indicated that the NJ equation also performed better than the unadjusted UPCR in subgroups defined by age and gender (all *P* values < 0.001) (shown in Supplementary Table 2).

**Fig. 3:**
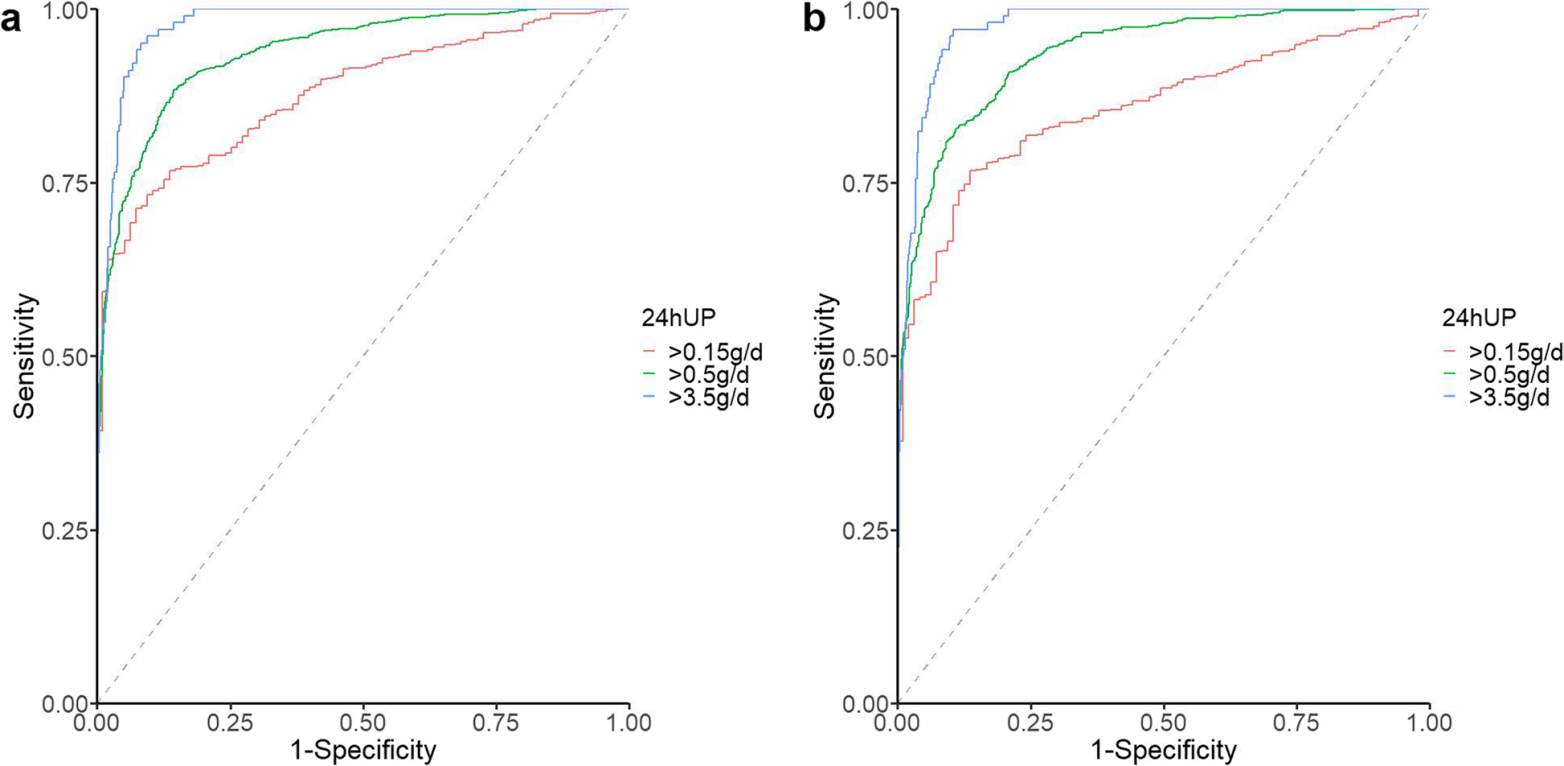
ROC analysis of the ability of unadjusted UPCR (**a**) and NJ equation (**b**) to predict 24 hUP above clinically relevant thresholds in the validation cohort

To further clarify the diagnostic value of NJ equation among situations of different degrees of proteinuria, we stratified the validation cohort by 24 hUP, with thresholds of 0.15 g, 0.5 g, and 3.5 g, respectively. A spot UPCR > 2.21 g/g represents threshold to correlate with a 24 hUP of > 3.5 g (positive likelihood ratio of 11.64, negative likely ratio of 0.05, accuracy of 0.91, and AUC of 0.98, shown in Supplementary Table 3). The ROC curves to detect 24 hUP values above thresholds of 0.15 g, 0.5 g and 3.5 g did not differ between unadjusted UPCR and e24hUP by NJ equation (Fig. 3). The areas under the ROC curves of unadjusted UPCR and 24 hUP by NJ equation were 0.88 vs 0.86 (*P* = 0.021), 0.94 vs 0.94 (*P* < 0.539), 0.98 vs 0.97 (*P* = 0.002), respectively in the validation cohort. (shown in Supplementary Table 3).

## Discussion

Quantification of urinary protein excretion is the cornerstone for the diagnosis and prediction of the outcome of glomerular diseases. The 24 hUP is the gold standard for measurement of protein excretion and is used in most clinical trials and clinical guidelines of nephrology, but sample collection is associated with several technical problems such as inaccurate collection and the potential spread of drug-resistant pathogens [2–5]. The measurement of UPCR is much easier than 24 hUP for patients and has become a popular alternative of the 24 hUP. Several previous studies showed a strong correlation between UCR and 24 hUP in patients with CKD or normal kidney function [3, 6, 17–19]. However, in our center, medical practitioners tend to refer to 24 hUP rather than UPCR when developing diagnosis and treatment strategies. In this study, we assessed the correlation between first-morning spot UPCR and 24 hUP from real-world data and confirmed a good correlation between the UPCR and 24 hUP in patients who visited our renal center. However, there was a systemic bias between UPCR and 24 hUP: the UPCR underestimated the 24 hUP (bias − 0.12 g/24 h). Thus, the UPCR cannot be directly compared with 24-h proteinuria in patients who underwent urine protein testing.

To improve the accuracy of 24 hUP prediction from a single spot UPCR, we developed an accurate prediction equation integrating demographic variables including age and gender. Several candidate models were derived and the model with the smallest RMSE (NJ equation) was chosen. The NJ equation also decreased the bias, IQR, and P30 compared with unadjusted UPCR, both in the training and validation cohort. After stratification by age and gender, the NJ equation still performed better than unadjusted UPCR.

Several previous studies also constructed models to predict 24 hUP based on a single spot UPCR [9, 12–14, 20, 21]. Chen et al. [12] constructed a 24 hUP prediction model with 1243 CKD patients in total based on morning spot UPCR, gender, age, body weight, and CKD stage. Compared with NJ equation, this model was inconvenient to apply, because bodyweight and CKD stage were not readily accessible in laboratory application. They found that spot UPCR can accurately predict 24 hUP in patients with lower 24 hUP (< 3 g/24 h). This was consistent with the results of our Bland-Altman plot, which indicated that NJ Equation performed better in patients with 24 hUP < 2 g/24 h than those with higher 24 hUP. By comparison, the model constructed by Hogan [9] was simple, with UPCR as the only variable. Hogan enrolled 226 adult CKD patients with biopsy-proven nephrotic diseases, the sample size of which was relatively small, especially when subgroup analyses were conducted. Moreover, the spot urine samples involved in Hogan’s study were random, while a prospective study showed that UPCR varies by sampling time during the day for the same patient [8]. In our study, we tried to construct a prediction model for laboratory application, where age and gender were easily accessible. Furthermore, the inclusion of gender and age did decrease the RMSE of the model. Thus, we constructed the prediction model only with UPCR, gender, and age, which could be automatedly applied in the laboratory test reports as a surrogate of UPCR for medical practitioners to refer to.

Here, we reported a 24 hUP prediction model from first-morning UPCR in our renal center, with a relatively large sample size (*n* = 5435). However, several limitations of the study should be addressed. First, due to laboratory application of NJ equation, confounding factors that have a potential influence on proteinuria were unavailable, nor involved in this study, including fever, diabetes, hypertension, urinary tract infection, indwelling urinary catheter, pregnancy, recipients of dialysis therapy, as well as renal transplant graft. Second, NJ equation was based on creatinine level, which should be used with caution in people with abnormally high or low levels of muscle mass. Third, although some published reports on proteinuric kidney disease were based on albuminuria or albumin-creatinine-ratio (ACR) [22, 23], we only report on total protein and not albumin excretion. According to the latest KDIGO guidelines [1], the albumin excretion rate and the ACR are not commonly used in nondiabetic forms of glomerular disease. In addition, the latest KDIGO guidelines further suggested that proteinuria in GN (separate from minimal change disease) is typically heterogeneous and consists of both albumin and other proteins. When a 24 hUP cannot be obtained, alternative method should be used. Furthermore, in clinical practice, albuminuria and 24 hUP were both routinely reported by the central laboratory at our hospital for each patient. Medical practitioners in our center prefer to refer to 24 hUP instead of albuminuria to determine total protein excretion. Fourth, this study was mainly conducted in the Han population, so the results may not represent what would be seen in a similar study in other ethnic background. Fifth, a previous meta-analysis revealed that spot UPCR has a utility as a screening test for proteinuria in patients with systemic lupus erythematosus, but these two tests showed poor agreement [24]. A spot urine PCR is reliable for 24 hUP prediction in patients with IgA nephropathy, but unreliable in patients with minimal change disease or Membranous glomerulonephritis with nephrotic syndrome [25]. Thus, pathological diagnosis might be a potential confounding factor in the prediction of 24 hUP based on spot UPCR. However, due to lack of diagnostic information by kidney biopsy in some patients, we failed to stratify the patients involved in this study by pathological diagnosis. Sixth, due to lack of routinely examined creatinine in 24-h urine collections, completement assessment of the collection was not conducted in term of at least 20 mg/kg creatinine in men (15 mg/kg in women).

## Conclusions

In conclusion, the NJ equation is more accurate for estimating 24 hUP than unadjusted UPCR, as shown in this large Chinese population, could be used for laboratory application in our renal center. This equation performed better in patients with 24 hUP < 2 g/24 h than those with higher 24 hUP. Despite the robustness of our model, further large-scale validation should be needed to establish a universal consensus.

## Supplementary Information

**Additional file 1: Supplementary Table 1.** Correlation analysis between UPCR and 24 hUP in the training cohort stratified by gender and age. **Supplementary Table 2.** Comparison of the UPCR and NJ Equations in Estimating Measured 24-h protein in the validation cohort stratified by age and gender. **Supplementary Table 3.** Threshold of spot UPCR to detect different degrees of 24 hUP in the validation cohort (*n* = 1624).

## Data Availability

The datasets used and/or analysed during the current study are available from the corresponding author on reasonable request.
