# Supplementary material for: An equation to estimate 24-hour total urine protein excretion rate in patients who underwent urine protein testing
Source: BMC Nephrol. 2022 Jan 29;23:49. doi: 10.1186/s12882-022-02673-2 (PMC8801121; doi:10.1186/s12882-022-02673-2)
Supplement: Supplementary file 1 — Additional file 1: Supplementary Table 1. Correlation analysis between UPCR and 24 hUP in the training cohort stratified by gender and age. Supplementary Table 2. Comparison of the UPCR and NJ Equations in Estimating Measured 24-h protein in the validation cohort stratified by age and gender. Supplementary Table 3. Threshold of spot UPCR to detect different degrees of 24 hUP in the validation cohort (n = 1624). [file 12882_2022_2673_MOESM1_ESM.docx]

Supplementary Table 1. correlation analysis between UPCR and 24hUP in the training cohort stratified by gender and age.

|  | Number | Pearson correlation coefficient | P |
| --- | --- | --- | --- |
| Overall | 3803 | 0.85 | <0.001 |
| Age |  |  |  |
| >48 years | 1799 | 0.85 | <0.001 |
| ≤48 years | 2004 | 0.86 | <0.001 |
| Gender |  |  |  |
| Male | 1987 | 0.87 | <0.001 |
| Female | 1816 | 0.84 | <0.001 |

Supplementary Table 2. Comparison of the UPCR and NJ Equations in Estimating Measured 24-hour protein in the validation cohort stratified by age and gender.

| Group | N | RMSE | |  | Bias | |  | IQR | |  | P30 | |
| --- | --- | --- | --- | --- | --- | --- | --- | --- | --- | --- | --- | --- |
|  |  | NJ equation | UPCR |  | NJ equation | UPCR |  | NJ equation | UPCR |  | NJ equation | UPCR |
| Age |  |  |  |  |  |  |  |  |  |  |  |  |
| ≤48y | 864 | 0.81 (0.71, 0.94) | 0.88 (0.76, 1.01) | | -0.021 (-0.036, -0.007) | 0.147 (0.131, 0.164) | | 0.32 (0.29, 0.35) | 0.37 (0.33, 0.41) | | 54.51 (51.30, 57.80) | 31.25 (28.10, 34.30) |
| >48y | 768 | 0.80 (0.70, 0.90) | 1.15 (0.99, 1.29) | | 0.01 (-0.007, 0.029) | 0.073 (0.054, 0.094) | | 0.37 (0.32, 0.44) | 0.64 (0.53, 0.75) | | 52.21 (48.90, 55.80) | 33.20 (30.10, 36.70) |
| Gender |  |  |  |  |  |  |  |  |  |  |  |  |
| Female | 777 | 0.76 (0.62, 0.90) | 1.00 (0.84, 1.17) | | 0.004 (-0.008, 0.019) | 0.064 (0.048, 0.080) | | 0.26 (0.23, 0.29) | 0.44 (0.38, 0.52) | | 51.87 (48.90, 55.50) | 32.69 (29.70, 36.10) |
| Male | 855 | 0.85 (0.74, 0.94) | 1.03 (0.92, 1.15) | | -0.023 (-0.047, -0.001) | 0.183 (0.160, 0.207) | | 0.43 (0.38, 0.49) | 0.47 (0.38, 0.54) | | 54.85 (51.40, 58.00) | 31.70 (28.60, 34.80) |

RMSE, root mean square error; UPCR, urine protein-creatinine ratio; IQR, interquartile range; P30, percentages of individuals that are within 30% difference.

Supplementary Table 3. Threshold of spot UPCR to detect different degrees of 24hUP in the validation cohort (n = 1624).

| 24hUP | >0.15g | |  | >0.5g | |  | >3.5g | |
| --- | --- | --- | --- | --- | --- | --- | --- | --- |
| Model | NJ equation | UPCR |  | NJ equation | UPCR |  | NJ equation | UPCR |
| Number | 1537 | 1537 |  | 933 | 933 |  | 102 | 102 |
| UPCR threshold (g/g) | 0.42 | 0.15 |  | 0.56 | 0.46 |  | 2.21 | 2.51 |
| Sensitivity | 0.71 | 0.77 |  | 0.88 | 0.83 |  | 0.95 | 0.97 |
| Specificity | 0.93 | 0.86 |  | 0.86 | 0.89 |  | 0.92 | 0.90 |
| Positive likelihood ratio | 4.98 | 7.06 |  | 8.57 | 8.50 |  | 11.64 | 9.28 |
| Negative likelihood ratio | 0.33 | 0.19 |  | 0.13 | 0.14 |  | 0.05 | 0.03 |
| Best Youden index | 0.64 | 0.63 |  | 0.74 | 0.72 |  | 0.87 | 0.87 |
| Accuracy | 0.73 | 0.77 |  | 0.87 | 0.85 |  | 0.92 | 0.90 |
| AUC | 0.88 | 0.86 |  | 0.94 | 0.94 |  | 0.98 | 0.97 |
